# The Sixteenth International Congress of Medicine

**Published:** 1908-12-12

**Authors:** 


### THE SIXTEENTH INTERNATIONAL CONGRESS OF MEDICINE.

A meeting of the National Committee for Great Britain and Ireland of the Sixteenth International Congress of Medicine was held at the rooms of the Medical Society of London on November 25 to consider the necessary organisation for the Congress of 1909, which will be held at Budapesth from August 29 to September 4. Dr. F. W. Pavy was in the chair, and there were present among others Sir William Church, Sir Dyce Duckworth, Sir Thornley Stoker, Sir Felix Semon, Dr. D. Ferrier, Mr. W. H. H. Jessop, Dr. F. W. Hewitt, and the honorary secretaries, Mr. D'Arcy Power and Dr. Clive Riviere. It was announced that all the hotel and lodging arrangements at Budapesth had been placed by the executive committee of the Congress in the hands of the International Sleeping Car Company, whose London offices are situated in Cockspur Street, Charing Cross. Members intending to visit Budapesth next year should make early application for rooms to the manager of the London branch. The South Eastern and Chatham Railway Company are considering the matter of through trains to the Congress at a reduction in the fare, but details would be published in due course. The question of the formation of a permanent international commission, to secure the continuity of policy in the international congresses, was discussed. In response to a letter from the general secretary as to the most convenient intervals between the congresses, a resolution was passed recommending that the international medical congresses should be held every four years instead of every three years, as hitherto.

